# Conduction Mechanisms in Resistance Switching Memory Devices Using Transparent Boron Doped Zinc Oxide Films

**DOI:** 10.3390/ma7117339

**Published:** 2014-11-12

**Authors:** Fu-Chien Chiu

**Affiliations:** Department of Electronic Engineering, Ming Chuan University, 5 De-Ming Rd., Gui-Shan, Taoyuan 33348, Taiwan; E-Mail: fcchiu@mail.mcu.edu.tw; Tel.: +886-3-350-7001 (ext. 3753); Fax: +886-3-359-3877

**Keywords:** boron doped zinc oxide, resistance switching, conduction mechanism, trap spacing, trap energy level

## Abstract

In this work, metal/oxide/metal capacitors were fabricated and investigated using transparent boron doped zinc oxide (ZnO:B) films for resistance switching memory applications. The optical band gap of ZnO:B films was determined to be about 3.26 eV and the average value of transmittance of ZnO:B films was about 91% in the visible light region. Experimental results indicated that the resistance switching in the W/ZnO:B/W structure is nonpolar. The resistance ratio of high resistance state (HRS) to low resistance state (LRS) is about of the order of 10^5^ at room temperature. According to the temperature dependence of current-voltage characteristics, the conduction mechanism in ZnO:B films is dominated by hopping conduction and Ohmic conduction in HRS and LRS, respectively. Therefore, trap spacing (1.2 nm) and trap energy levels in ZnO:B films could be obtained.

## 1. Introduction

Zinc oxide (ZnO) has a wide direct bandgap energy of 3.37 eV, which makes it transparent in visible light and is a promising candidate for blue and ultraviolet light emitting devices (LEDs) and lasers [1]. In general, ZnO with a wurtzite structure is an unintentional n-type semiconductor due to the deviation from stoichiometry. The background free electrons essentially arise from the shallow donor levels associated with the presence of intrinsic defects such as oxygen vacancies and/or zinc interstitials [1]. For higher conductivity n-type ZnO films, intentional n-type doping can be established through the substitution of group III elements (B, Al, Ga, and In) on the Zn sites or group VII elements (F and Cl) on the O sites [1]. After doping of group III elements, ZnO becomes attractive to replace indium tin oxide (ITO) as the transparent conducting electrodes in liquid crystal displays or solar cell devices because of abundant raw material, simple manufacturing process with low cost, low synthetic temperature, competitive optical and electrical properties, nontoxicity and stability in plasma [2]. One interesting feature of ZnO is the ability to bandgap tuning by its alloying with magnesium oxide (MgO, *E_g_* ~7.7 eV) or cadmium oxide (CdO, *E_g_* = 2.3 eV). Namely, bandgap energy of 3.9 eV (Mg*_x_*Zn_1−*x*_O, *x* = 0.33) can be achieved by doping with Mg^2+^, while Cd^2+^ decreases the bandgap energy to 2.99 eV (Cd*_y_*Zn_1−*y*_O, *y* = 0.07) [3]. In addition, the large exciton binding energy of 60 meV of ZnO is of interest to achieve excitonic stimulated emission for the realization of low-threshold lasers at room temperature and even higher temperatures. Recently, ZnO-based diluted magnetic semiconductors showed ferromagnetism in ZnO by doping with boron or a transition metal, which appears promising to achieve practical Curie temperature for future spintronic devices [4,5].

In this work, a metal-oxide-metal structure was fabricated and investigated using transparent boron doped zinc oxide (ZnO:B) films for the application of resistance random access memory (RRAM). RRAM is one of the novel memories, which promises to overcome the physical limitations of traditional Flash memory for the next generation nonvolatile memory applications [6]. The attractiveness of RRAM technology is its good compatibility with the complimentary metal-oxide-semiconductor (CMOS) process [7], which means that the scaling merit may be continued in terms of the low power consumption which will then bring a strong cost-competitiveness to RRAM. In addition, the merits of RRAM include high switching speed, high operation durability, small cell size, simple cell structure, multi-state switching and three-dimensional architecture [8,9,10,11].

## 2. Results and Discussion

**Figure 1:**
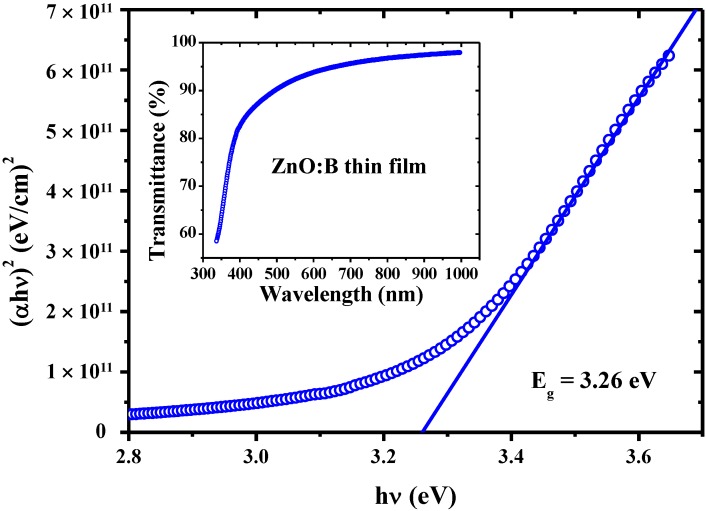
Optical band gap calculated by the (αhν)^2^-energy of the transmittance curve. Inset shows the optical transmittance of boron-doped ZnO (ZnO:B) films.

The transmittance spectra of boron-doped ZnO films in the wavelength range of 340–1000 nm are shown in the inset of Figure 1. The average transmittance of boron-doped ZnO film is about 90.9% in the visible region (400–700 nm). To identify the optical absorption coefficient (*α*) for ZnO:B films, Lambert’s law is used [12].

(1) $$\alpha =\frac{1}{d}\ln(\frac{1}{T})$$

where *d* is the film thickness and *T* is the transmittance. In a direct-transition semiconductor material, the absorption coefficient *α* is correlated to the optical band gap by the following equation [12].

(2) $${\left(\alpha h\nu \right)}^{2}=A(h\nu -E_{g})$$

where *h* is Planck’s constant; ν is the frequency of the incident photon; *A* is a constant that depends on the mobility of the electrons and holes in the material; *E_g_* is the optical band gap. Hence, the optical band gap of ZnO:B films is determined by Tuac’s plot, namely, the linear part of the (*αhν*)^2^ curve is extrapolated toward the energy *hν* axis at (*αhν*)^2^ = 0. The determined band gap of ZnO:B films is about 3.26 eV, as shown in Figure 1.

**Figure 2:**
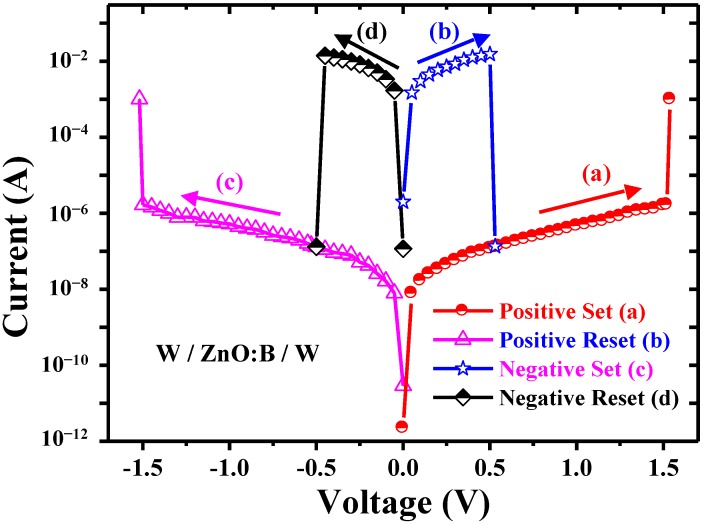
Typical nonpolar I-V switching characteristics of W/ZnO:B/W memory cells.

Typical current-voltage (*I-V*) switching characteristics in the W/ZnO:B/W memory cells are shown in Figure 2. Symmetric *I-V* curves are observed in these memory cells. The resistance ratio of high resistance state (HRS) to low resistance state (LRS) is about of the order of 10^5^ for a compliance current (*I*_comp_) of 1 mA at room temperature. In this work, the memory cells were initially at LRS. When a voltage sweeps from 0 to either positive or negative bias without a current compliance, the device current decreases suddenly at a reset voltage (*V*_reset_) or a reset current (*I*_reset_) and the device is switched from a LRS to an HRS. This event is named the “RESET” process. On the other hand, by re-sweeping the voltage in the positive or negative side with 1 mA *I*_comp_, an abrupt increase of the current takes place at a set voltage (*V*_set_) or a set current (*I*_set_). The *V*_set_ triggers the memory cell from an HRS to a LRS, which is named the “SET” process. Note that the *I*_comp_ of 1 mA was set to prevent the permanent breakdown of the memory devices during the SET process in this work. Conversely, no *I*_comp_ was set in the RESET process due probably to failed switching. Based on the *I-V* switching characteristics in Figure 2, the ZnO:B RRAM device was free of electroforming in this work. Both free of electroforming and the initial low resistance state may be attributed to the high conductivity of ZnO:B films which can be deposited by *dc* magnetron sputtering. According to the *I-V* switching behaviors, the *V*_set_ ~1.5 V is larger than *V*_reset_ ~0.5 V, and the *I*_set_ ~1 × 10^−6^–2 × 10^−6^ A is smaller than the *I*_reset_ ~1 × 10^−2^–2 × 10^−2^ A. Both the SET and RESET processes were independent of voltage polarity in this work. Hence, the resistance switching (RS) in W/ZnO:B/W structure is nonpolar. The nonpolar RS shows the coexistence of unipolar and bipolar RS characteristics. This clear reversible and reproducible switching between LRS and HRS can be observed at temperatures ranging from 298 to 398 K. Reports show that nonpolar resistance switching is also found in some oxide films, such as MgO and indium gallium zinc oxide (IGZO) [13,14].

**Figure 3:**
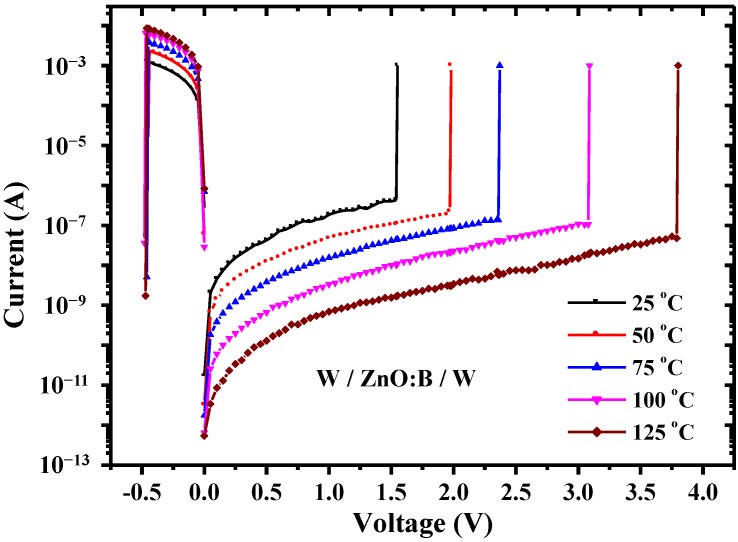
Temperature dependence of *I-V* switching characteristics in W/ZnO:B/W memory devices.

**Figure 4:**
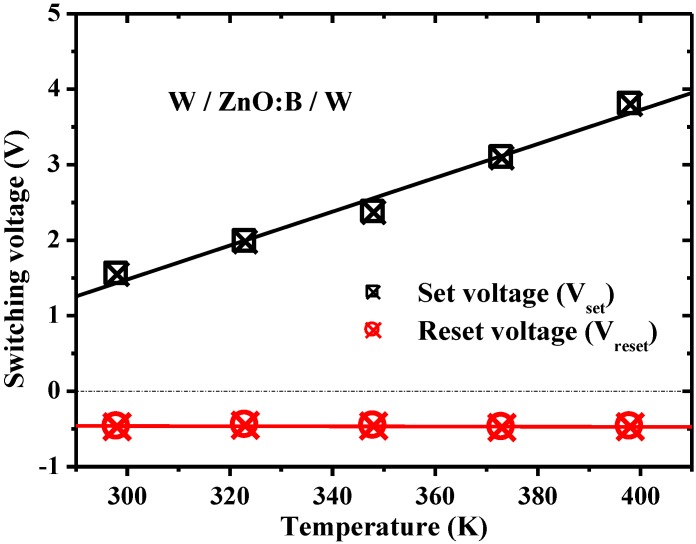
Temperature dependence of SET and RESET voltages on W/ZnO:B/W memory devices.

**Figure 5:**
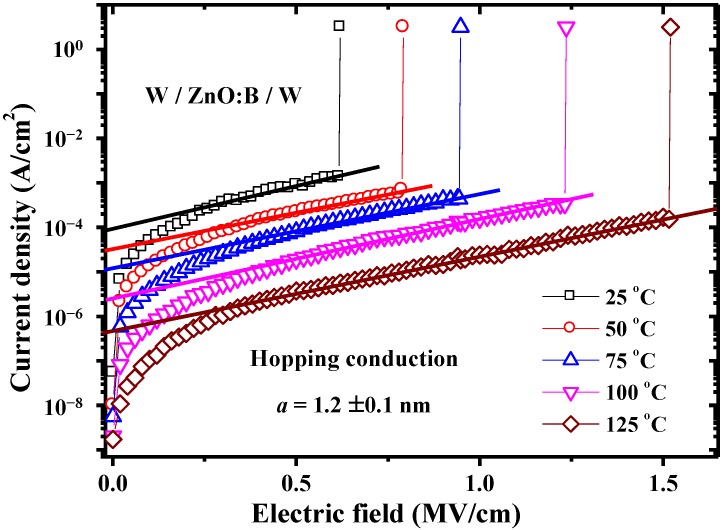
Experimental data and simulation curves of hopping conduction in high-resistance state in W/ZnO:B/W memory devices.

**Figure 6:**
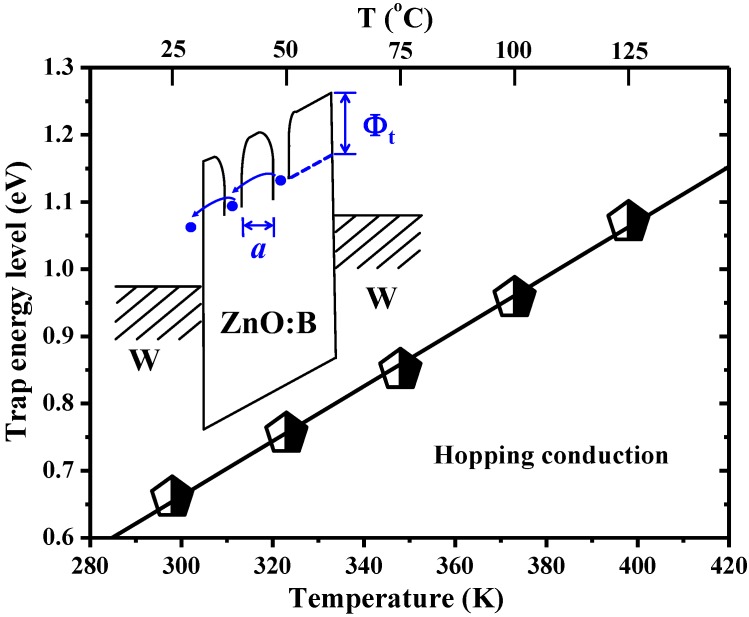
Temperature dependence of trap energy levels in the high-resistance state. Inset shows the band diagram of hopping conduction in W/ZnO:B/W memory devices.

Since conduction mechanisms are generally dependent on temperature in different ways, measuring the conduction current as a function of temperature provides very useful information on the constitution of the conduction currents [15]. Figure 3 shows the temperature dependence of *I-V* characteristics both in HRS and LRS at temperatures ranging from 298–398 K under the condition of 1 mA compliance current. Obviously, *V*_set_ is enhanced at the elevated temperature, whereas, *V*_reset_ is almost constant, as shown in Figure 4. According to the temperature dependence of *I-V* characteristics in HRS, an interesting device behavior was discovered, namely, a lower current level was obtained at a higher temperature. This discovery is very different from the normal *I-V* characteristics in oxide devices in which the higher the temperature the larger the current. To identify the dominant conduction mechanism, electric current simulations and typical plots of relations between current density (*J*) and electric field (*E*) were adopted. The simulation results show that experimental data match the theory of hopping conduction very well when the electric field is larger than about 0.35 MV/cm, as shown in Figure 5. Hence, the dominant conduction mechanism in the W/ZnO:B/W structure is the hopping conduction in HRS. In hopping conduction, the carrier energy is lower than the maximum energy of the potential barrier between two trapping sites, as depicted in the inset of Figure 6. Thus, the carrier transportation in ZnO:B takes place with the aid of a tunneling effect in HRS. The hopping conduction can be expressed as [15]:

(3) $$J=qanv\text{ exp}\left[\frac{qaE}{kT}-\frac{{\text{Φ}}_{t}}{kT}\right]$$

where *q* is the electronic charge; *a* is the hopping distance (*i.e*., mean spacing between trap sites); *n* is the electron concentration in the conduction band; *v* is the frequency of thermal vibration of electrons at trap sites; *E* is the applied electric field; *T* is the absolute temperature; *k* is the Boltzmann’s constant; and Φ*_t_* is the energy level from the trap states to the bottom of the conduction band (*E_C_*). According to Equation (3), the value of *qa/kT* can be extracted by the slope of the linear part of ln(*J*) * versus* *E* at each temperature. The term “*a*” means the hopping distance between traps, namely, “*a*” is the trap spacing. Hence, the trap spacing in ZnO:B is determined to be 1.2 ± 0.1 nm according to Figure 5. Similarly, the value of Φ*_t_* can be extracted by the intercept of the linear part of ln(*J*) *versus* *E* at each temperature. Therefore, the temperature dependence of trap energy levels in ZnO:B films can be obtained. As shown in Figure 6, the trap energy level increases with temperature. This implies that the defects in ZnO:B films with deeper trap levels are activated by the elevated temperature. These deeper-level traps activated at higher temperatures result in the exponential decrease in current density accordingly. This phenomenon is also observed in MgO RRAM devices [13]. A report showed that a deep donor level of 0.85 eV was obtained at high temperature (~400–460 K) in ZnO:B films prepared by the sol-gel method using a spin coating technique [16]. In this work, the deep-trap energy levels in HRS at 298 and 398 K are about 0.66 and 1.07 eV, respectively. Apparently, these deep traps play the key role in the current conduction in ZnO:B films in HRS. Both MgO and ZnO:B devices show the conductivity reduction at high temperature in HRS, which comes from the deeper-level traps activated at elevated temperature. These defects with deeper trap levels should be introduced during the deposition processes and could be the origin of nonpolar switching in which both unipolar and bipolar switching behaviors can coexist. Consequently, not single-level but multiple-level traps can be found in MgO and ZnO:B devices. Note that the current density through the oxides will be raised at elevated temperatures for devices with traps of single-level. For the case of single-level traps in oxides, such as non-doped ZnO [17], the nature of temperature enhanced current density in HRS is very different from the case of multiple-level traps in boron-doped ZnO in which the current density is reduced by temperature. Basically, the switching properties of B-doped ZnO, non-doped ZnO, and MgO devices are not the same. The non-doped ZnO devices show bipolar switching which is different from the nonpolar switching in MgO devices. Furthermore, the B-doped ZnO devices are free of electroforming, whereas both non-doped ZnO and MgO devices need a high electroforming voltage to initiate switching behavior. Through the substitution of boron atoms on the zinc sites in this work, one electron could be ionized to become a conduction electron in the ZnO film at room temperature. As a consequence, high conductivity n-type ZnO films were obtained, which resulted in the phenomena of initial low-resistance state and free-of-electroforming. In the meantime, the multiple-level traps were introduced during the boron doping process. These multiple-level traps are the origin of current reduction at high temperature in HRS in B-doped ZnO devices. In addition, compared to MgO devices, the ZnO:B devices may have a lower switching voltage and a larger leakage current, because the bandgap of ZnO:B (~3.3 eV) is much smaller than that of MgO (7–8 eV). Aside from the consideration of bandgap, the energy barrier at the metal/oxide interface is also a dominant factor for the evaluation of switching voltage and leakage current. In a word, the switching characteristics are not only dependent on the oxide materials but also dependent on the metal electrodes and their interfacial properties. In this work, the dominant current conduction mechanism was not related to the electrode-oxide interface but to the oxide bulk because hopping conduction was one of the bulk-limited conduction mechanisms. The bulk-limited conduction mechanism depends only on the properties of the oxide itself [15]. The energy band diagram of carrier transport in hopping conduction is shown in the inset of Figure 6.

**Figure 7:**
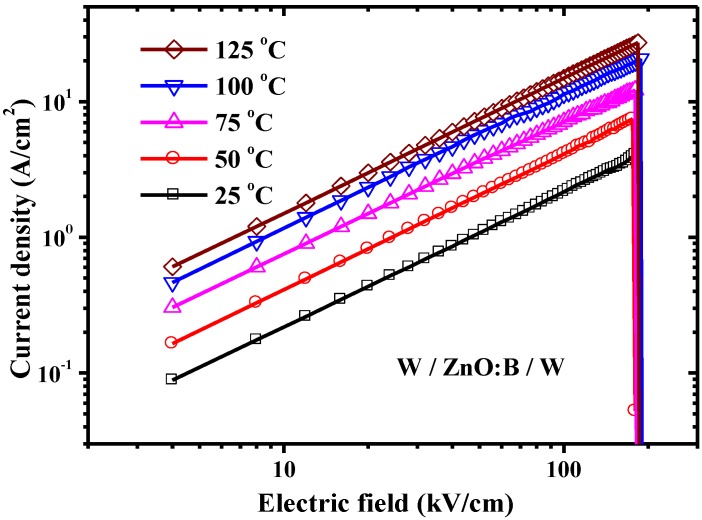
Experimental data and simulation curves of Ohmic conduction in low-resistance state in W/ZnO:B/W memory devices.

**Figure 8:**
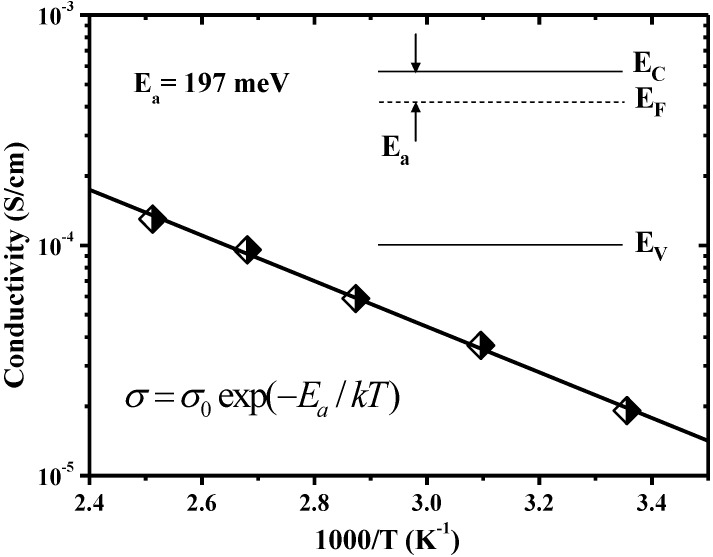
Temperature dependence of electrical conductivity in low-resistance state. Inset shows the location of Fermi level in Ohmic conduction in W/ZnO:B/W memory devices.

In LRS, the current density increases with temperature. The *J-E* curves are shown in Figure 7 in a double-logarithmic plot. According to the temperature dependence of *I-V* switching characteristics shown in Figure 3, the HRS/LRS resistance ratio is about of the order of 10^5^ at 298 K and its value increases with temperature in the W/ZnO:B/W structure. Figure 7 shows that a linear relationship exists between current density and electric field, which matches the Ohmic conduction very well because the slope of the linear function in each temperature is very close to one. The Ohmic conduction can be expressed as [15];

(4) $$J=\sigma E=q\mu N_{C}E\text{ exp}\left[\frac{-(E_{C}-E_{F})}{kT}\right]$$

where *σ* is electrical conductivity; *μ* is electron mobility; *N_C_* is the effective density of states of the conduction band; and *E_F_* is the Fermi energy level; the other terms are as defined above. Figure 8 indicates that a negative linear relationship exists between electrical conductivity and inverse temperature in LRS. According to the Arrhenius plot, *E_F_* in LRS is determined to be about 197 meV below *E_C_*, as shown in the inset of Figure 8. The data is close to the result in Ref. [16] in which a shallow donor level of 160 meV exists in ZnO:B films. The increasing trend of current density with temperature in LRS shown in Figure 7 presents a negative slope in Figure 8. This thermally activated conduction is a semiconductor-like behavior which is different from the thermally depressed conduction in metal. The semiconductor-like behavior in LRS can be also found in devices with non-doped ZnO as well as MgO [13,17]. Interestingly, the metal-like behavior in LRS exhibits in some metal oxides, such as NiO [18]. Due to Joule heating in the conductive filament in NiO films, the resistance in LRS increases with temperature. Although the reasons are not clear, the semiconductor-like or metal-like behavior in LRS is perhaps related to the oxide materials. In this work, the dominant conduction mechanism in ZnO:B films was hopping conduction and Ohmic conduction in HRS and LRS, respectively. The hopping conduction is associated with carrier tunneling among traps in the oxide bulk. Aside from the hopping conduction, the Ohmic conduction is also a bulk-limited conduction mechanism [15]. A report indicated that the resistance switching in metal/oxide/metal structures is generally attributed to the formation and rupture of conductive filaments in oxide bulk [7]. Based on the analyses of conduction mechanisms, both hopping conduction in HRS and Ohmic conduction in LRS are bulk-limited conductions. Deep-level traps participate in hopping conduction in HRS, whereas shallow-level traps participate in Ohmic conduction in LRS. This suggests that the resistance switching mechanism in the W/ZnO:B/W structure is not the interface-type but the filament-type. The filament-type switching mechanism is associated with the oxide traps in B-doped ZnO, non-doped ZnO and also MgO films. The formation of conductive filaments corresponds to the Ohmic conduction through these filaments in LRS with the aid of shallow-level traps in oxide films. Conversely, the rupture of conductive filaments corresponds to hopping conduction through the oxide film in HRS with the aid of deep-level traps.

## 3. Experimental Section

In this work, boron-doped ZnO (ZnO:B) films of 25 nm were deposited on W/Ti/SiO_2_/Si substrates using *dc* magnetron sputtering in Ar ambient at room temperature. The *dc* power was 100 W and the power density was 0.6 W/cm^2^. The flow rate of argon was 50 standard cubic centimeters per minute (sccm). The working pressure during deposition was 5 mtorr. In this work, the deposited ZnO:B films were amorphous based on the analysis of X-ray diffraction (XRD) (not shown here). To achieve the W/ZnO:B/W RRAM devices, a tungsten (W) top electrode was deposited by *dc* magnetron sputtering with a round area of 3.14 × 10^−4^ cm^2^ patterned by the metal shadow mask. In this work, 10% titanium (Ti) was added into the W target to improve the contact adhesion and to relax the mechanical stress. The W thickness is 500 nm. Note that the boron doping concentration of ZnO:B films was about 0.8 wt%. The resistivity of ZnO:B films assessed by a four-probe method was about 9.5 × 10^−3^Ω-cm. The electrical characteristics of the fabricated RRAM devices were measured by an Agilent 4156C semiconductor parameter analyzer (Agilent Technologies, Hachioji, Japan). All the measurements were performed under dark conditions.

## 4. Conclusions

In conclusion, the optical properties and current transports in W/ZnO:B/W memory devices were studied. The band gap of ZnO:B films is about 3.26 eV with an average transmittance of 91% in the visible light region. Experimental results showed that the dominant conduction mechanism in ZnO:B films was hopping conduction and Ohmic conduction in HRS and LRS, respectively. Accordingly, the trap spacing and trap energy levels in ZnO:B films could be obtained. The current conduction mechanisms in HRS and LRS are related to deep-level traps and shallow-level traps, respectively. The ZnO:B films with high transparency show potential to be used in the development of nonvolatile memories.
